# P-1299. Epidemiology of Invasive Meningococcal Disease in the United States: Review of Recent Data and Identified Risk Factors

**DOI:** 10.1093/ofid/ofae631.1480

**Published:** 2025-01-29

**Authors:** Jessica Presa, Daniel Spitz, Paul Palmer, Vincenza Snow, Kathleen Dooling

**Affiliations:** Pfizer, Inc., Collegeville, Pennsylvania; Pfizer Inc, Collegeville, Pennsylvania; Pfizer Vaccine Medical Development, Scientific & Clinical Affairs , Collegeville PA, Collegeville, PA; Pfizer Vaccines, Collegeville, PA; Pfizer Inc., Collegeville, Pennsylvania

## Abstract

**Background:**

Although relatively rare, invasive meningococcal disease (IMD) is distinguished by its unpredictable epidemiology, rapid clinical progression, and often severe or fatal outcomes. IMD risk peaks in infants and young children, with secondary peaks in adolescents/young adults and sometimes older adults. Overall IMD has been declining in the United States and elsewhere since the year 2000, with historic lows during the COVID-19 pandemic; however, widespread post-pandemic rebounds stress the ongoing public health threat of IMD.

In the United States, IMD is a notifiable condition tracked by the Centers for Disease Control and Prevention (CDC) in part using the National Notifiable Diseases Surveillance System (NNDSS). A comprehensive enhanced meningococcal disease surveillance (EMDS) program was implemented in 2015.

Presa_Figure

**Figure:**
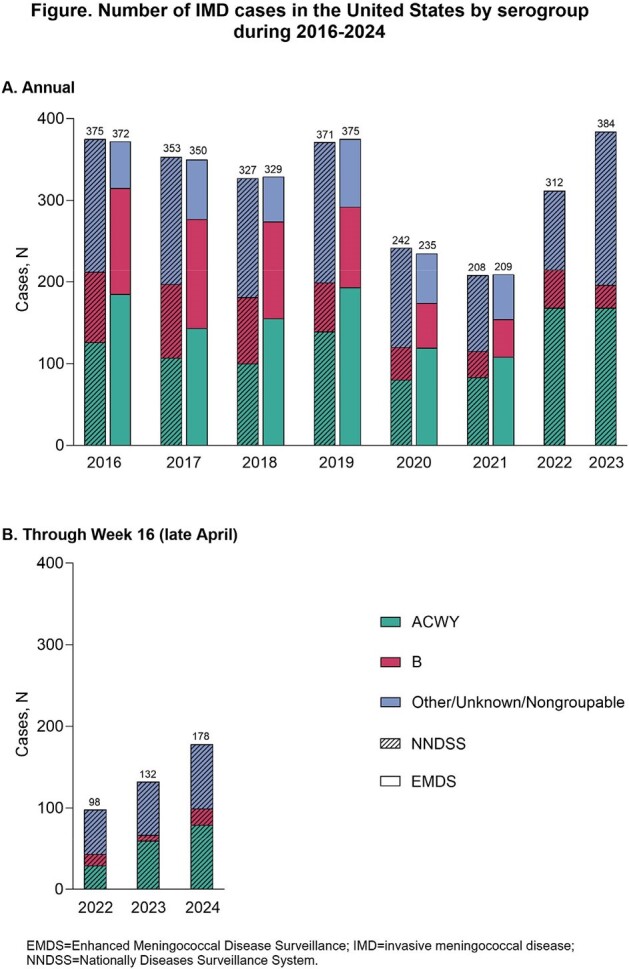

N/A

**Methods:**

We compiled annual NNDSS and EMDS data and searched published literature for analyses of US IMD data to review recent IMD epidemiology and associated risk factors.

**Results:**

The NNDSS and EMDS reported similar numbers of IMD cases during 2016‒2021, with EMDS providing more updated serogroup information (Figure). NNDSS data indicate more cases in 2023 than in any of the 7 years prior, with this rebound intensifying thus far in 2024; the CDC estimates there will be more cases in 2024 than the 422 seen in 2023 (35% serogroup Y). Serogroup Y ciprofloxacin and/or penicillin resistance rose from 22.8% in 2019 to 65.4% in 2021. Surveillance data and additional analyses point to increased IMD incidence associated with college vs no college attendance (1.6- to 3.1-fold during 2016‒2019), Black vs White race (1.1- to 2.4-fold, 2016‒2020), Medicaid vs commercial insurance (1.8- to 3.0-fold, 2016‒2019), homelessness vs non-homelessness (13.8- to 30.2-fold, 2016‒2019), men having vs not having sex with men (5.4-fold, 2015‒2016), and HIV vs no HIV infection (6.2-fold; 2009‒2019).

**Conclusion:**

US IMD is rebounding after major COVID-associated declines. Established risk factors (eg, smoking, sharing food, dormitory living) and related behaviors (eg, vaping, overnight camp attendance) may be influencing IMD surges as post-COVID socialization increases. Comprehensive understanding of US epidemiology is crucial for optimizing vaccination strategies. Funding: Pfizer.

**Disclosures:**

**Jessica Presa, MD**, Pfizer: Employee|Pfizer: Stocks/Bonds (Public Company) **Daniel Spitz, PharmD**, Pfizer Inc: Employee **Paul Palmer, PhD**, Pfizer Inc: Employee **Vincenza Snow, MD**, Pfizer: Employee|Pfizer: Stocks/Bonds (Public Company) **Kathleen Dooling, MD**, Pfizer: Employee|Pfizer: Stocks/Bonds (Public Company)

